# Endo-Aortic Clamping with the IntraClude^®^ Device in Minimally Invasive Total Coronary Revascularization via Left Anterior Thoracotomy (TCRAT)

**DOI:** 10.3390/jcm13195891

**Published:** 2024-10-02

**Authors:** Christian Sellin, Hilmar Dörge, Parwis Massoudy, Andreas Liebold, Robert Balan

**Affiliations:** 1 Department of Cardiothoracic Surgery, Heart-Thorax Center, Klinikum Fulda, University Medicine Marburg, Campus Fulda, 36043 Fulda, Germany; christian.sellin@klinikum-fulda.de (C.S.); doerge@klinikum-fulda.de (H.D.); 2 Department of Cardiac Surgery, Klinikum Passau, 94036 Passau, Germany; parwis.massoudy@klinikum-passau.de; 3 Department of Cardiothoracic and Vascular Surgery, Ulm University Hospital, 89081 Ulm, Germany; andreas.liebold@uniklinik-ulm.de; 4 I.O.S.U.D., George Emil Palade University of Medicine, Pharmacy, Science and Technology of Targu Mures, 540139 Targu Mures, Romania

**Keywords:** minimally invasive cardiac surgery, coronary artery bypass grafting, CABG, TCRAT, aortic endo-clamp

## Abstract

Minimally invasive, sternum-sparing total coronary revascularization in multivessel disease via left anterior mini-thoracotomy (TCRAT) was introduced recently. Intra-aortic balloon occlusion is a conceivable option to avoid manipulation of the ascending aorta, to reduce the risk of stroke and to be able to treat patients with severe calcifications and unfavorable aortic anatomies. **Background/Objectives**: The aim of our study was to show that the use of the IntraClude^®^ device, as part of minimally invasive coronary artery bypass grafting (CABG) via left anterior mini-thoracotomy, is feasible. **Methods**: From May to December 2023, CABG via left anterior mini-thoracotomy on cardiopulmonary bypass and cardioplegic arrest was successfully performed in 20 patients (17 male, 67.6 ± 8.2 (51–82) years). All patients had significant coronary artery disease (three-vessel: *n* = 6; two-vessel: *n* = 11; one-vessel: *n* = 3) with indication for surgical revascularization. The mean EuroScore2 was 2.6. **Results**: All patients successfully underwent minimally invasive CABG using endo-aortic balloon occlusion. A total of 43 distal anastomoses (2.2 ± 0.6 (1–3) per patient) were performed by using left internal artery mammary (*n* = 20) and radial artery (*n* = 14) for grafting the left anterior descending (*n* = 19), circumflex (*n* = 15) and right (*n* = 6) coronary artery. There was no hospital mortality, no stroke, no myocardial infarction or repeat revascularization. A total of 15 out of 20 patients left hospital within 8 days after surgery. **Conclusions**: TCRAT by using the IntraClude^®^ device is feasible without compromising surgical principles while avoiding the external manipulation of the ascending aorta. The use of intra-aortic balloon occlusion instead of transthoracic clamps further reduces the invasiveness of the procedure.

## 1. Introduction

Coronary artery bypass grafting (CABG) remains the most robust therapies for coronary revascularization in multivessel coronary disease [1] and is therefore recommended as a first-line therapy for complex multivessel coronary disease [2]. Full median sternotomy is still the standard approach for the majority of CABG procedures worldwide [3]. In order to reduce the invasiveness of conventional CABG with full median sternotomy and the associated limitations with regard to physical activity, quality of life and pain, minimally invasive procedures, e.g., robotic endoscopic CABG were developed, but are currently used only in specialized centers and on highly selected patients [4,5]. Babliak et al. [6] proposed a new surgical approach for complete coronary revascularization in multivessel coronary artery disease via left anterior mini-thoracotomy (TCRAT). This technique was further developed by Dörge et al. [7] as a novel, less invasive routine concept of CABG avoiding sternotomy. Using proven surgical principles like cardiopulmonary bypass (CPB), aortic cross clamping, cardioplegic cardiac arrest and standard anastomotic techniques, the procedure can be applied on unselected patients with promising early results [7,8]. In order to further reduce the invasiveness of the TCRAT technique by avoiding a thoracic incision for transthoracic aortic cross clamping and the external manipulation of the ascending aorta, especially in cases of severe calcifications and strongly elongated ascending aorta, we used an intra-aortic occlusion device (IntraClude^®^ intra-aortic balloon device, Edwards Lifesciences Perimount, Irvine, CA, USA). The IntraClude^®^ device is a triple lumen catheter that occludes and vents the ascending aorta when the balloon is inflated. The device’s central lumen allows the delivery of cardioplegia to arrest the heart. This report describes the surgical technique and summarizes our initial experience.

## 2. Materials and Methods

From May to December 2023, 20 elective patients with coronary artery disease underwent minimally invasive CABG using CPB and cardioplegic cardiac arrest.

In all patients, both the indication for surgical revascularization according to current guidelines and the identification of target vessels were subjected to an interdisciplinary heart team discussion [2,9]. Emergency patients (same-day catheterization and operation) were not accepted. Relevant information as part of our internal quality assurance documentation was retrospectively extracted from our database. A descriptive analysis was performed, and data are presented as means (±standard deviation) or percentages.

### 2.1. Ethical Standards

This study has been proven by the local ethic committee (TEMP742237-evBO). It was performed in accordance with the ethical standards laid down in the Declaration of Helsinki from 1964 and its later amendments.

### 2.2. Preoperative Evaluation

All patients underwent a CT angiography from the supra-aortal vessels to the groin in addition to the standard institutional preoperative examinations to screen the ascending aorta, the aortic arch and major arterial branches, especially the iliac and femoral vessels, for atherosclerotic disease and anatomical abnormalities.

The user manual of the IntraClude^®^ device describes the absolute and relative contraindications for use:

Absolute:

- Aneurysm of the ascending aorta.
- Severe aortic regurgitation.

Relative:

- Moderate to severe peripheral or aortic atherosclerosis.
- History of thoracic trauma.

Patients presenting any of the above mentioned abnormalities were not considered for endo-aortic clamping.

Furthermore, the quality and usability of radial artery (RA) were evaluated with ultrasound, Doppler ultrasound, and the Allen test.

### 2.3. Anesthesia

Standard cardiac anesthesia techniques (i.v. sufentanil 0.5 µg/kg/h, etomidate 0.25 mg/kg, pancuronium 0.1 mg/kg, sevoflurane, propofol 3 mg/kg/h) were used for the induction and maintenance of anesthesia. All patients were intubated with a single-lumen endotracheal tube. Invasive monitoring was performed with standard arterial and venous lines. In all patients, transesophageal echocardiography (TOE) was performed to assist positioning the guidewires for femoral arterial and venous cannulation, advancing and positioning the balloon catheter and monitoring heart function during the operation.

### 2.4. Surgical Technique

The operations were performed in the supine position.

Endoscopic radial artery harvesting was performed either using a reusable retractor (Bisleri Model, Karl Storz, Tuttlingen, Germany) and a bipolar radiofrequency vessel sealing system (LigaSure, Medtronic, Minneapolis, MN, USA), or the Vasoview Hemopro Endoscopic Vessel Harvesting System (Getinge AB, Rastatt Germany). RA was stored in a preservation solution using iron chelators (TiPROTEC, Dr. Franz Köhler Chemie GmbH, Bensheim, Germany). Through an anterior mini-thoracotomy of about 8 cm in the fourth intercostal space, the chest was opened and a retractor (Small Thoracotomy Retractor, Delacroix-Chevalier, Paris, France) was inserted. The left internal mammary artery (LIMA) was identified in the fourth intercostal space. After careful preparation medially beyond the pedicle, two clips were placed, and the mammary artery (LIMA) was divided. The free pedicle was then harvested under direct surgical vision as a pedicle beyond the origin of left mammary vein using long conventional surgical instruments (35 cm DeBakey forceps and 15 cm electrocautery blade) and a special retractor (MIDAccess IMA Retractor, Delacroix-Chevalier, Paris, France). Then, 400 U/kg heparin was administered intravenously. Peripheral arterial cannulation was performed via right axillary artery (16/18 Fr OptiSite Arterial Perfusion Cannula; Edwards Lifesciences, Irvine, USA). Percutaneous venous cannulation was achieved through the common femoral vein via the Seldinger technique. A venous cannula (23 Fr Bio-Medicus, Medtronic, Minneapolis, USA) was placed in the right atrium, guided by TOE. In case of a body surface area greater than 2.0 m^2^, an additional venous cannula was inserted in the jugular vein (15/17 Fr Bio-Medicus, Medtronic, Minneapolis, USA) to enhance the venous return. Another option to enhance the venous return was to opt for the percutaneous placement, through the common femoral vein of the venous Smart Canula^®^ (SmartCanula LLC, Leusane, Switzerland). Vacuum-assisted venous return was routinely used during CPB to improve heart decompression. During CPB, patients were kept normothermic.

Alternatively, if the groin arterial vessels had a diameter of at least 7 mm, we opted for a percutaneous arterial cannulation with a 21 French or 23 French EndoReturn^®^ (Edwards Lifesciences, Irvine, CA, USA) arterial sheet.

### 2.5. Endoclamp

**Figure 1:**
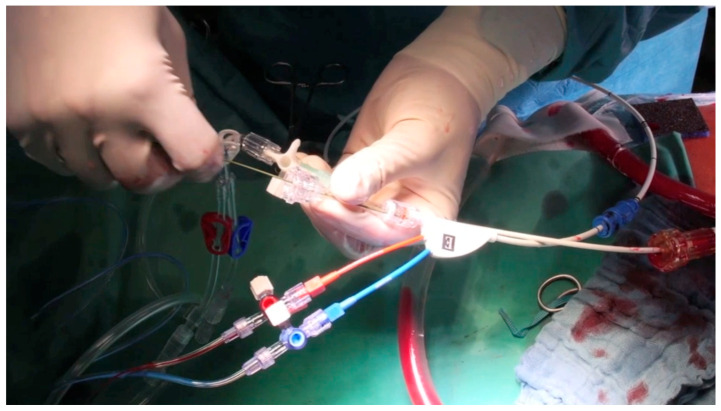
IntraClude^®^ device—triple lumen balloon catheter transfemoral insertion. The catheter features aortic balloon occlusion, cardioplegia administration, pressure monitoring, de-airing and venting.

**Figure 2:**
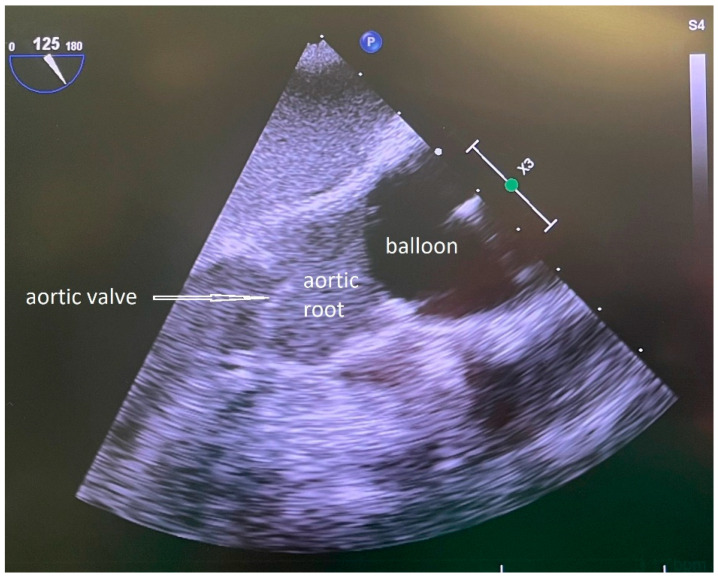
TOE control of the filled balloon of the IntraClude^®^ device placed in the ascending aorta. Tip of the arrow pointing to the aortic valve.

An arterial sheath (16 Fr Adelante^®^ MAGNUM, Oscor Inc., Palm Harbor, CA, USA) was inserted percutaneously via the left common femoral artery to gain access for the IntraClude^®^ device, a 10.5 Fr (3.5 mm), triple-lumen, 100 cm long catheter (Figure 1). Guided by TOE, the balloon of the IntraClude^®^ device was placed in the ascending aorta above the coronary ostia. After opening the pericardium, inspection of the coronary vessels was performed, with the identification of the target vessels and an epicardial pacer was placed on the diaphragmatic side of the heart. After electrical induction of ventricular fibrillation, the balloon was gradually filled with saline until the aorta was occluded (Figure 2). During this critical part of the operation, care was taken that the pressure remained identical in the left and right arterial pressure line, in order to ensure that the balloon was placed proximally from the Truncus brachicephalicus and did not occlude, or partially compromise cerebral perfusion. During the inflation of the balloon TEE was performed, carefully inspecting the distance between the aortic valve and the endo-clamp balloon, the tip of the balloon being preferably placed at a distance of 2–3 cm from the aortic valve, leaving the aortic bulb free. In order to confirm complete occlusion of the ascending aorta, two parameters were used: the pressure of the aortic root, proximally from the inflated balloon, and the pressure in the balloon (preferably higher than 400 mmHg). Total occlusion was achieved in the moment the pressure in the aortic root dropped significantly compared to the systemic blood pressure. TEE and bilateral pressure monitoring were routinely used during the whole procedure, in order to immediately identify a potential dislocation or rupture of the endo-clamp balloon.

Antegrade cold blood cardioplegia (modified Buckberg solution, Dr. Franz Köhler Chemie GmbH, Bensheim, Germany) was delivered through the central perfusion lumen of the catheter. Cardioplegia was repeated after each peripheral anastomosis. Balloon pressure and aortic root pressure were continuously monitored. The balloon pressure was kept at target levels around 400 mmHg at all times.

After the heart was arrested and decompressed, left pulmonary veins and inferior vena cava were encircled with tapes. By pulling on these tapes in combination with rotation of the heart, all coronary territories could be reached by reducing the distance from skin incision of the small anterior lateral mini-thoracotomy to coronary arteries to less than 10 cm. In this way, coronary artery target sites could be exposed for manual palpation and assessment. Moreover, a stable exposition for preparation, performing of the anastomoses, and conventional knotting of all anastomotic sutures were possible. Coronary anastomoses were performed with standard anastomotic technique of running 8-0 polypropylene sutures and with usual coronary surgical instruments. The LIMA was anastomosed as an in situ graft to the left anterior descending artery (LAD). The second graft (either radial artery or the sapheneous vein) was anastomosed to the LIMA as a composite T-graft or Y-graft. This anastomosis was also accomplished during cardiac arrest. Thereafter, further peripheral coronary anastomoses were performed using the length of the respective second graft.

After deflating the balloon, the IntraClude^®^ device was removed. The arterial sheath was removed after the completion of CPB. All bypass grafts were checked using transit time flow measurement (QuickFit TTFM, Medistim, Deisenhofen, Germany). The arterial vascular closure was accomplished using the Proglide vascular closure system (Perclose ProGlide™, Abbott, Chicago, IL, USA) or the MANTA vascular closure device (Teleflex, Wayne, PA, USA). After decannulation, the venous cannulation site was compressed using a 2.0 compressive skin suture to the puncture site and the placement of a SafeGuard (MIN Medical, Vienna, Austria).

## 3. Results

The mean patient age was 67.6 ± 8.2 years. Overall, 17 out of 20 patients had multivessel coronary disease; 15 patients had symptoms according to New York Heart Association (NYHA) functional classification in class 3. The baseline and cardiovascular characteristics are given in Table 1.

The average number of distal anastomoses was 2.2 ± 0.6, and the maximum number of anastomoses was 3. The LAD coronary artery territory was grafted in 19, the RCX territory in 15 and the RCA territory in 6 out of 20 patients. All coronary targets preoperatively recommended by the heart team could be anastomosed. Operative data are given in Table 2.

There was no hospital mortality, no myocardial infarction, no repeat revascularization and no stroke. Postoperative new atrial fibrillation occurred in three patients. In summary, the complication rate was very low. Fifteen patients left the hospital within 8 days after the operation. The postoperative adverse events and outcomes are given in Table 3.

## 4. Discussion

The aim of this study was to show that the use of the IntraClude^®^ device for intraoperative aortic occlusion, application of cardioplegia and venting of the left ventricle, as part of minimally invasive CABG via anterior mini-thoracotomy, is technically feasible. The main area of application of the IntraClude^®^ device so far has been mitral valve surgery [10,11]. The avoidance of manipulation of the ascending aorta using the IntraClude^®^ device is likely to be the main benefit regarding the prevention of stroke, especially in patients with severe calcifications of the ascending aorta. In comparison to minimally invasive CABG via left anterior mini-thoracotomy described by Babliak et al. [6] and further developed by Dörge et al. [7], no additional thoracic incision is needed for transthoracic aortic clamp, which can lead to a reduction in pain and possible bleeding complications.

In the past, minimally invasive CABG often required a compromise between surgical trauma and complete revascularization. Recently, some studies [7,8] have shown that complete coronary revascularization (meaning targets indicated in heart team discussions could be treated intraoperatively) was achieved in over 95% of cases by performing minimally invasive CABG via anterolateral mini-thoracotomy. In this study, a complete coronary revascularization rate of 100% was achieved. Using the IntraClude^®^ device did not lead to any compromise in coronary revascularization regarding the rotation of the heart required for the operation technique. In our series, the IntraClude^®^ device showed a stable position in the ascending aorta without balloon dislodgement and with sufficient venting function of the left ventricle.

To use the IntraClude device, a femoral arterial access is required. This can be achieved either via a side arm of the dedicated perfusion cannula (EndoReturn^®^, Edwards Lifesciences, Irvine, CA, USA) or via direct percutaneous access. In this study, a 16 Fr sheath was inserted percutaneously using a Perclose Proglide^®^ suture-mediated closure system, or the MANTA vascular closure device (Teleflex, Wayne, PA, USA). Therefore, its use in patients with calcific disorders of the abdominal aorta, iliac and femoral vessels is limited. Possible resulting vascular complications such as bleeding, hematomas, perforations or local dissection did not occur in our study. In addition, lesions of the aorta resulting from the advancement and inflation of the balloon catheter, including dissection and rupture, are conceivable [12,13]. Furthermore, the diameter of the ascending aorta should not exceed ≤ 20 mm and ≥40 mm according to the instructions for use of the IntraClude^®^ device. Although this type of surgery usually takes more time than that needed for routine CABG via median full sternotomy, the use of the balloon might possibly help to distinctly shorten the procedure [14]. Careful balloon handling as well as wire skills are advisable to ensure a safe procedure. Good patient selection, a highly competent team, the use of an appropriate insertion technique and intraoperative TOE imaging, and careful pressure monitoring are key for a successful procedure [10,15,16].

The ambition of reducing the invasiveness of cardiopulmonary bypass becomes more and more relevant in the era of minimally invasive cardiac surgery. Introducing extracorporeal circulation over peripheral vessels was a first step followed by the application of an endo-aortic balloon in minimally invasive mitral valve surgery. We herein describe our approach to further reduce the invasiveness of surgical treatment of coronary artery disease by introducing the endo-aortic balloon to CABG surgery. With the use of the IntraClude^®^ device, the sometimes technically demanding external clamping of the aorta and the necessity of introducing a cardioplegia canula into the ascending aorta are eliminated, thus overall reducing the risks of potential complications and developing a technically less challenging and safer way to perform minimally invasive CABG.

## 5. Limitations

Furthermore, this study has some limitations. The lack of previous data on this topic as well as the off-label use of endo-aortic clamping during aorto-coronary bypass surgery has to be considered. The IntraClude^®^ device is a tool which can be used safely in high-volume centers with the necessary expertise, yet the learning curve with this device is steep. This study describes a pilot cohort of patients operated on with this modified technique, and further investigations on larger patient groups have to be performed to support our findings.

## 6. Conclusions

In conclusion, minimally invasive total coronary revascularization via left anterior thoracotomy using the IntraClude^®^ device is feasible while avoiding the manipulation of the ascending aorta. This can be an important step in the treatment of patients with severe calcifications of the ascending aorta, in patients with anatomically unfavorable conditions and in the prevention of strokes. With growing experience, IntraClude^®^ has the potential to replace the transthoracic clamp in minimally invasive CABG surgery, for selected patients.

## Figures and Tables

**Table 1:** Baseline parameters (*n* = 20).

Age (Years)	67.6 ± 8.2 (51–82 Years)
BMI	28.1 ± 3.3 (22.1–36.2)
Hypertension	19 (95%)
Diabetes mellitus	4 (20%)
Cardiovascular parameters	
Symptoms according to New York Heart Association:	
NYHA 1	0 (0%)
NYHA 2	4 (20%)
NYHA 3	16 (80%)
NYHA 4	0 (0%)
1-vessel disease	3 (15%)
2-vessel disease	11 (55%)
3-vessel disease	6 (30%)
Recent myocardial infarction	6 (30%)
EF (%)	49.7 ± 12.5 (10–60)
EuroScore2	2.6 ± 3.1

Data are presented as mean (±standard deviations) or absolute values (percentage %). BMI: body mass index; EF: left ventricle ejection fraction.

**Table 2:** Operative data.

Conduits Used	
LIMA	20 (100%)
RA	14 (70%)
SVG	4 (20%)
Revascularization territory of	
LAD	19 (95%)
RCX	15 (75%)
RCA	6 (30%)
Number of distal anastomoses	2.2 ± 0.6
1	2 (10%)
2	13 (65%)
3	5 (25%)
Length of surgery (minutes)	265 ± 78
CPB time (minutes)	116 ± 30
Aortic cross-clamp time (minutes)	68 ± 24

Data are presented as mean (±standard deviations) or absolute values (percentage %). LIMA: left internal mammary artery; SVG: saphenous vein graft; RA: radial artery; LAD: left anterior descending artery; RCX: ramus circumflexus; RCA: right coronary artery; CPB: cardiopulmonary bypass.

**Table 3:** Postoperative adverse events and outcomes.

Adverse Events	
Low cardiac output	0 (0%)
Myocardial infarction	0 (0%)
Revision due to bleeding	1 (5%)
Dialysis	0 (0%)
Delir	0 (0%)
Pneumonia	0 (0%)
New onset of atrial fibrillation	3 (15%)
Superficial wound infection	0 (0%)
Stroke	0 (0%)
Outcome parameters	
Time on ICU	2.1 ± 1.2
≤1 day	7 (35%)
In-hospital stay (days)	8.5 ± 3.1
≤8 days	15 (75%)
In-hospital mortality	0 (0%)

Data are presented as mean (±standard deviations) or absolute values (percentage %). ICU: intensive care unit.

## Data Availability

The original contributions presented in the study are included in the article, further inquiries can be directed to the corresponding author.
